# A Deep Learning-Guided Ensemble Empirical Mode Decomposition Method for Single-Channel Fetal Electrocardiogram Extraction

**DOI:** 10.3390/s26072037

**Published:** 2026-03-25

**Authors:** Xiaojian Xu, Yifan Zhang, Yufei Rao, Yinru Xu, Yang Gao, Huating Tu

**Affiliations:** 1 School of Health Science and Engineering, University of Shanghai for Science and Technology, Shanghai 200093, China; 243352502@st.usst.edu.cn (X.X.);; 2 College of Medical Instruments, Shanghai University of Medicine & Health Sciences, Shanghai 201318, China; 3 Shanghai Key Laboratory of Intelligent Sensing and Detection Technology, School of Mechanical and Power Engineering, East China University of Science and Technology, Shanghai 200237, China

**Keywords:** fetal electrocardiogram (FECG), single-channel FECG extraction, ensemble empirical mode decomposition (EEMD), one-dimensional convolutional neural network (1D CNN), intrinsic mode functions (IMFs), non-invasive home fetal monitoring

## Abstract

The fetal electrocardiogram (FECG) is critical for assessing fetal cardiac electrophysiology and detecting fetal distress and arrhythmias. Single-channel abdominal electrocardiogram (AECG) enables home-based monitoring but faces challenges posed by weak fetal signals, maternal interference, and the lack of spatial information. Ensemble Empirical Mode Decomposition (EEMD) is suitable for nonstationary AECG signals but relies on accurate selection of intrinsic mode functions (IMFs). In this study, a deep learning-guided method was proposed: a one-dimensional convolutional neural network (1D CNN) scored and selected EEMD-derived IMFs, followed by maternal QRS template subtraction and secondary EEMD purification to achieve automatic FECG extraction. Leave-one-subject-out (LOSO) cross-validation was performed on 15 simulated cases and 5 ADFECGDB records, yielding a mean AUC of 0.9282 ± 0.0189 for the IMF classifier. On the independent DaISy and NIFEA arrhythmia datasets, the proposed CNN-2×EEMD method achieved correlation coefficients of 0.94–0.96, F1-scores of 0.8372–0.9565 for fetal R-peak detection, and SNR improvements of 13.39–15.88 dB. This method outperformed conventional automatic selection methods and matched the performance of manual selection. Ablation studies validated the optimal network design and IMF selection strategy, while complexity analysis (0.08 GFLOPs, 2.24 ms latency) confirmed its suitability for real-time wearable deployment.

## 1. Introduction

Non-invasive fetal electrocardiogram (FECG) monitoring is a vital clinical tool for evaluating fetal cardiac electrophysiology during late pregnancy and delivery. The bandwidth of the FECG is typically 5–100 Hz, with the dominant energy of the fetal QRS complex concentrated at approximately 8–45 Hz. FECG provides precise timestamps for each cardiac cycle and complete QRS–T morphological details, enabling the early identification of fetal distress, arrhythmias, and other abnormalities, thereby helping to reduce the incidence of adverse perinatal outcomes [1,2]. Nevertheless, in abdominal electrocardiogram (AECG) signals, the amplitude of the fetal component is typically only 1/5 to 1/10 that of the maternal electrocardiogram (MECG), or even lower. The FECG is therefore severely contaminated by strong maternal interference, electromyographic noise, respiration-induced baseline drift, power-line interference, and other instrumental disturbances. Although multichannel systems can enhance signal separation via spatial filtering, they are bulky, costly, and impractical for long-term home monitoring. In comparison, single-channel acquisition is characterized by simple hardware, a compact size, low cost, and superior wearability, making it highly suitable for home-based fetal surveillance. This configuration can be used to improve maternal compliance and expand the accessibility of FECG monitoring [3,4]. Accordingly, the efficient extraction of FECG from single-channel AECG is not only essential for addressing existing technical bottlenecks but also fundamental for realizing portable and widely accessible home fetal monitoring, thereby improving the timeliness and reliability of fetal health assessment [5,6].

Classical signal processing techniques, including Independent Component Analysis (ICA), Principal Component Analysis (PCA), adaptive filtering, wavelet transform, and template coherent averaging subtraction, deliver satisfactory performance in multichannel FECG extraction. However, under single-channel acquisition conditions, the absence of spatial information, combined with weak fetal signal components and nonstationary noise, significantly complicates maternal–fetal signal separation. Behar et al. reported that single-channel approaches such as template subtraction (TS), adaptive filtering, and echo state networks (ESNs) exhibited performance degradation under limited spatial information, particularly in the presence of strong maternal interference or high noise levels [7]. ICA-based blind source separation relies on statistical independence and requires multichannel recordings, rendering it inapplicable to single-channel AECG signals. Adaptive filtering enables online updating for non-invasive FECG extraction [8], yet it heavily depends on high-quality maternal reference signals that are difficult to acquire reliably. Wavelet-based methods suppress noise through multiresolution decomposition but are sensitive to threshold selection and may distort the morphology of fetal QRS complexes under low-signal-to-noise ratio (SNR) conditions [9]. Although TS effectively removes maternal QRS complexes [10], it demonstrates limited robustness against high levels of background noise and temporal overlap between maternal and fetal cardiac waveforms.

Data-driven decomposition methods have emerged as promising alternatives for single-channel FECG extraction. Empirical Mode Decomposition (EMD) and Ensemble Empirical Mode Decomposition (EEMD) are well suited for nonlinear and nonstationary AECG signals, as they do not require predefined basis functions [11,12,13]. Dash and Nath combined EMD with wavelet decomposition for fetal QRS extraction [14]; however, their selection of intrinsic mode functions (IMFs) was based on fixed frequency criteria, which limits the robustness of the method under low-SNR conditions. Zhang and Guo applied EMD combined with coherent averaging [13], but threshold-based selection carries the risk of retaining maternal residual components or discarding weak fetal ECG content. Liu and Luan introduced an EEMD-based method that uses energy and correlation coefficients for IMF selection [15], yet manual intervention was still required. Barnova et al. integrated ICA, recursive least squares (RLS), and EEMD [16], but manual IMF selection reduced the degree of automation and limited clinical practicability. Despite their adaptive decomposition capability, EEMD-based approaches still rely on heuristic rules or manual experience for IMF selection, which results in inconsistent performance across diverse clinical scenarios.

Although existing studies made important progress in system platform architecture and semi-automatic algorithm workflow, the core algorithmic bottleneck in single-channel fetal ECG extraction, namely the automatic selection of IMFs after EEMD decomposition, remained insufficiently resolved. In this study, a one-dimensional convolutional neural network (1D CNN) was adopted for IMF classification instead of Transformer or Long Short-Term Memory (LSTM) architectures. EEMD-derived IMFs exhibit local and quasi-periodic impulse patterns, and their discriminative features mainly lie in QRS morphology and periodicity rather than long-range dependencies. 1D CNNs capture these local characteristics through translation-invariant convolution with fewer parameters and provide high computational efficiency that is critical for biomedical applications [17,18]. In contrast, Transformers require excessive computational resources, while LSTMs are prone to gradient vanishing and slow convergence [19,20]. Conventional machine learning methods rely on handcrafted features that cannot adequately describe the nonlinear and nonstationary characteristics of fetal QRS complexes, especially under low-SNR conditions [21,22,23]. Studies that focus on the semantic discrimination of individual EEMD-derived IMFs and systematically integrate deep learning into single-channel FECG extraction remain limited [24,25], and this gap further motivated the proposed hybrid framework.

This study presented a deep learning-guided method for single-channel FECG extraction. This approach replaced manual component selection in conventional EEMD-based methods with a data-driven IMF classification module. First, EEMD decomposes single-channel AECG into multiple IMFs. A trained 1D CNN then analyzes each IMF and outputs fetal-related probabilities to automatically identify fetal-dominant components, thereby eliminating subjective component selection and enhancing robustness. Building on this module, EEMD decomposition, intelligent IMF selection, maternal QRS template coherent averaging subtraction, and a second EEMD-based purification step were integrated into a sequential multi-stage signal processing pipeline. Unlike conventional hybrid architectures requiring manual intervention for IMF selection, the proposed framework completely eliminates this subjective manual procedure. Previous approaches retain operator-dependent heuristic rules for component identification; in contrast, the proposed method uses a physiologically guided 1D CNN to classify IMFs based on fetal probability scores. This data-driven strategy transformed the conventional semi-automatic pipeline into a fully automated system without compromising clinical interpretability. The contribution of this study extends beyond algorithmic integration, providing a reproducible standard for single-channel FECG extraction using adaptive decomposition.

## 2. Materials and Methods

### 2.1. Method Validation Design

To rigorously evaluate the effectiveness, robustness, and clinical generalizability of the proposed single-channel FECG extraction method, a two-stage validation protocol was designed and implemented in this study. First, a mixed dataset was constructed using 15 high-fidelity simulated signals with accurate fetal R-peak ground-truth annotations (noise augmented to SNR = 10 dB) and 5 real clinical recordings from the Abdominal and Direct Fetal Electrocardiogram Database (ADFECGDB). A leave-one-subject-out (LOSO) cross-validation scheme was adopted to train and evaluate the IMF classification model, thereby preventing inter-subject data leakage and ensuring an objective assessment.

**Figure 1:**
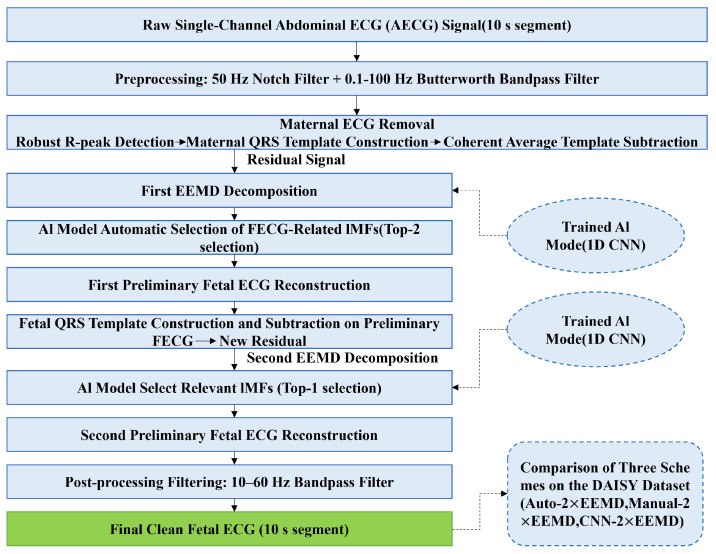
The Overall Flowchart.

Second, short single-channel recordings from the Database for the Identification of Systems (DaISy), which were not used for training, were introduced as an independent external test set. Under identical preprocessing and maternal template subtraction settings, a head-to-head comparison was conducted between the proposed 1D CNN-guided IMF selection method (CNN-2×EEMD), a fixed-heuristic automatic selection method (Auto-2×EEMD), and manual visual selection (Manual-2×EEMD, used as a manual visual reference). Performance was evaluated using multiple metrics, including waveform fidelity, fetal R-peak detection accuracy, and signal-to-noise ratio improvement, in conjunction with visual inspection of the reconstructed waveforms. The overall procedure is shown in Figure 1.

### 2.2. Dataset Description

To balance data diversity and training efficiency under a limited sample size, 15 single-channel abdominal ECG signals were generated. By adjusting key parameters, including maternal heart rate, fetal heart rate, and the fetal-to-maternal amplitude ratio, these signals covered clinical scenarios ranging from normal pregnancy to low-SNR conditions with weak fetal components. This design ensured sufficient diversity and difficulty for learning fetal characteristics, while LOSO was enabled to reduce overfitting and provide an objective assessment of generalization. Moreover, the chosen sample size limited the computational burden and reproduced the physiological property that fetal amplitudes are markedly lower than maternal amplitudes, providing reliable supervised labels for IMF classification under conditions close to clinical practice. Because high-quality public AECG recordings are scarce and often lack accurate fetal R-peak annotations, they could not directly support supervised deep learning training. Therefore, the 15 signals were generated using an H2-3000F maternal–fetal ECG simulator, and precise fetal R-peak annotation was performed on a 500 Hz sampling grid. The fetal ECG simulator (Xuzhou Mingsheng Electronic Technology Co., Ltd., Xuzhou, China) integrates four 18,650 lithium batteries and a dedicated power management module to ensure stable, low-interference operation. It supports 12 V DC charging and provides dual-channel outputs for maternal-only and mixed maternal–fetal ECG signals. Maternal and fetal parameters such as heart rate, R-wave amplitude, and signal intensity can be flexibly adjusted to simulate various clinical conditions. This simulator is widely used for algorithm development and validation of maternal–fetal ECG monitoring systems. However, systematic discrepancies exist between simulated and real clinical recordings, which may weaken model generalization. In typical simulators, the fetal-to-maternal amplitude ratio is fixed between 1:1.17 and 1:16, with only Gaussian noise and simple baseline drift added. Real abdominal ECG signals show dynamic amplitude fluctuations and complex non-Gaussian noise, including electromyographic interference and irregular baseline wander. Such domain mismatch may degrade model performance in practical applications. To alleviate this issue, random amplitude jitter was introduced during simulation to replicate physiological variability, and real clinical noise segments were incorporated to augment the training data. Based on the parameters listed in Table 1, 15 synthetic datasets were generated, covering maternal heart rates of 60–120 bpm, fetal heart rates of 110–160 bpm, and variable fetal–maternal amplitude ratios. These cases included both normal and low-SNR conditions to better represent real clinical signals.

In addition, five clinical recordings were selected from ADFECGDB (sampling rate: 1000 Hz) to enhance the model’s generalization to real-world clinical data [26,27]. Together with the 15 simulated cases spanning extreme physiological parameter settings and a LOSO strategy, this design mitigated the limited number of clinical samples and supported an objective evaluation of generalization. ADFECGDB provides simultaneous recordings of direct fetal ECG reference leads and multiple abdominal channels. This study focuses on single-channel FECG extraction, so one abdominal channel was selected as the sole input for both training and inference. During IMF classifier training, the direct fetal lead was used only to detect fetal R-peaks and label the IMFs decomposed from the corresponding abdominal signal. For final inference, the direct fetal lead was excluded entirely; only the selected abdominal channel served as input, consistent with the single-channel paradigm. To ensure consistent processing of simulated and clinical data and reduce the effects of noise and frequency interference on subsequent EEMD decomposition and model training, a standardized preprocessing pipeline was applied to all signals used for IMF classifier training.

**Figure 2:**
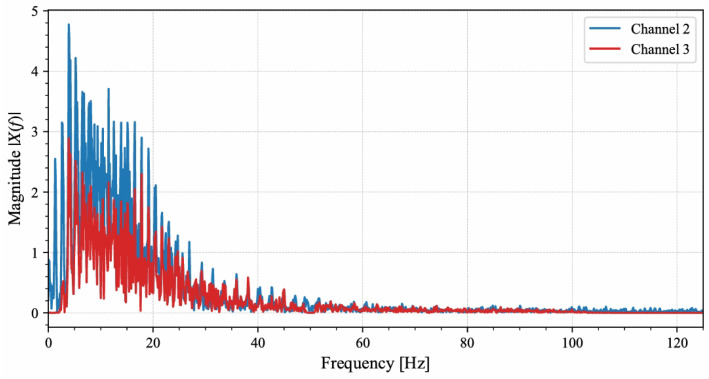
The Frequency Domain Distribution of Channels 2 and 3 in the DaISy Dataset.

**Figure 3:**
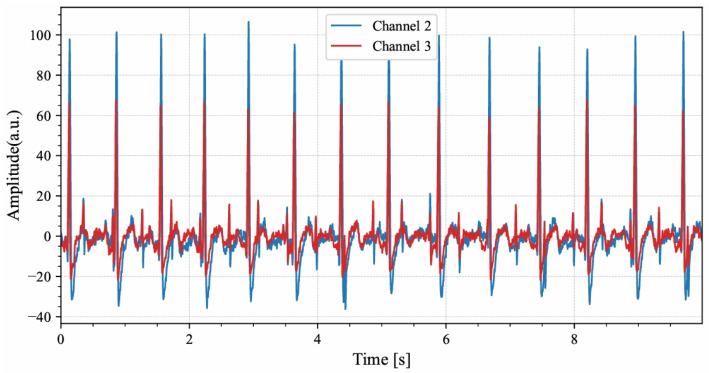
The Time Domain Comparison of Channels 2 and 3 in the DaISy Dataset (Ordinate: a.u. (arbitrary unit), representing the relative signal amplitude): The blue curve represents the AECG of Channel 2, and the red curve represents the AECG of Channel 3.

To further examine generalization on a fully independent external dataset, short single-channel recordings from DaISy that were not involved in training were used. DaISy was sampled at 250 Hz; each record was 10 s long, and five abdominal channels were available, though fetal R-peak annotations were not provided [28]. Based on preliminary signal analysis, channels 2 and 3 were selected for validation. As shown in Figure 2, these channels exhibited concentrated energy within the characteristic 8–45 Hz band. Figure 3 shows that these channels contained fetal QRS complexes with sharper rising and falling edges and more stable RR intervals than other channels, which facilitated the construction of reliable pseudo-references. Fetal R-peak locations were manually verified on a beat-by-beat basis, and ambiguous samples were excluded, providing a robust basis for head-to-head comparisons among the three IMF selection strategies.

### 2.3. Preprocessing

#### 2.3.1. Sampling Rate Unification

The simulated data were resampled from 500 Hz to 1000 Hz to ensure consistent processing with the ADFECGDB dataset. The abdominal signals in ADFECGDB were originally sampled at 1000 Hz and thus required no resampling. Resampling was implemented using a polyphase filtering method with an upsampling factor of 2 and a downsampling factor of 1, as expressed in Equation (1):

(1) $$x_{1000}\left(n\right)=\sum_{k=-\infty }^{\infty }x_{500}\left(k\right)\cdot h\left(2n-k\right)$$

where h(k) is the unit impulse response of the polyphase filter, which ensures accurate mapping of the signal frequency components.

#### 2.3.2. Signal Filtering

A 50 Hz IIR notch filter was used to remove power-line interference. A fourth-order Butterworth bandpass filter (0.1–100 Hz) was then applied via zero-phase forward–backward filtering (filtfilt) to avoid phase distortion. The 0.1 Hz cutoff attenuated baseline wander without excessive waveform distortion, while the 100 Hz upper limit preserved diagnostically relevant frequency content: maternal ECG components extend to 100 Hz and fetal QRS complexes reach approximately 80–90 Hz.

#### 2.3.3. Noise Augmentation (Simulated Training Data)

**Figure 4:**
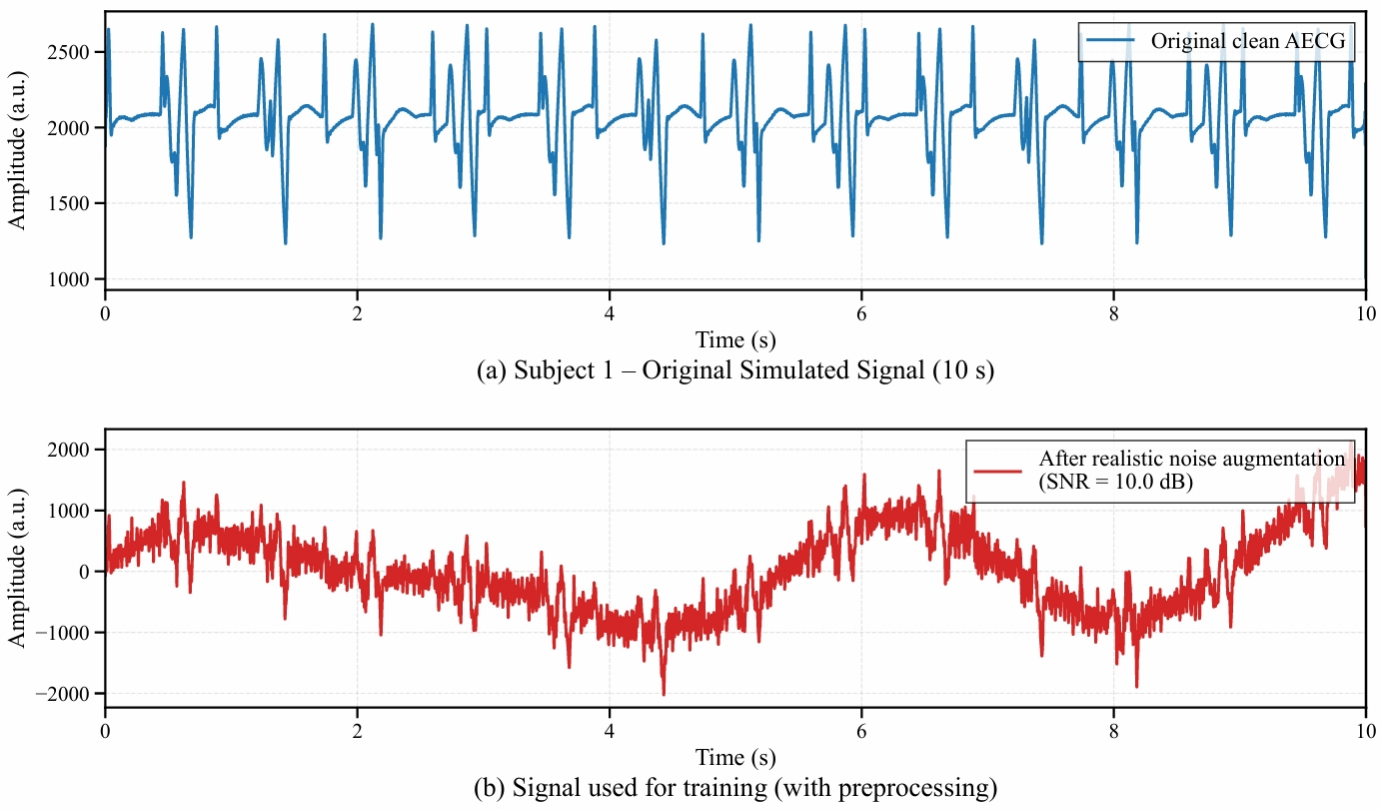
A Comparison of Simulated AECG Signals Before and After Noise Enhancement for the First Simulation Case: (**a**) The Original Noise-Free Simulated AECG Signal; (**b**) The AECG Signal with Superimposed Muscle Interference and Baseline Drift (SNR = 10 dB).

To better match the SNR distribution observed in clinical recordings, the simulated AECG was augmented with synthetic EMG interference and baseline wander, and the overall SNR was controlled to 10 dB after augmentation (Figure 4). In clinical practice, after contamination by EMG and baseline drift, the fetal signal SNR typically fell within 8–12 dB; thus, 10 dB was a representative value for common medium-to-low-SNR scenarios. This setting avoided insufficient noise at high SNR (e.g., >15 dB), which may reduce model generalization, while also preventing extremely low SNR (e.g., <5 dB) where fetal components are fully masked and meaningful features become difficult to learn. Consequently, the training data remained both challenging and informative, and the learned IMF discrimination capability was closer to practical clinical conditions [29,30]. The noise synthesis procedure was as follows.

EMG interference simulation: Gaussian white noise was generated and then bandpass-filtered at 20–150 Hz to obtain EMG-like components. Its amplitude was scaled to 0.1 times the standard deviation of the original AECG signal to mimic high-frequency interference caused by muscle activity.

Baseline wander simulation: Two low-frequency sinusoids at 0.2 Hz and 0.33 Hz were generated and summed, with an amplitude set to 0.18 times the standard deviation of the original AECG signal, to reproduce slow baseline fluctuations induced by respiration and posture changes.

Noise was added following the energy superposition principle. By precisely adjusting the amplitude ratio between the two noise sources, the overall SNR of the augmented signals was strictly maintained at 10 dB. The noise-augmented signals were processed using exactly the same preprocessing pipeline as the original signals: 50 Hz notch filtering followed by bandpass filtering. This ensured a consistent training data distribution, avoided additional bias introduced by preprocessing, and provided a realistic and standardized noise environment for model training.

### 2.4. Ensemble Empirical Mode Decomposition

After the above preprocessing, power-line interference and broadband noise were reduced, and baseline wander was partially attenuated, providing a cleaner input for subsequent separation of fetal and maternal ECG components [31]. Next, EEMD was used for adaptive decomposition. The nonstationary AECG signal was decomposed into IMFs with different time scales and frequency characteristics, providing interpretable units for subsequent IMF classification. By introducing noise-assisted decomposition, EEMD could alleviate mode mixing in nonstationary signals [16]. The decomposition was expressed as follows:

(2) $$x\left(t\right)=\sum_{i=1}^{8}IMF_{i}\left(t\right)+r\left(t\right),$$

where x(t) denotes the input AECG signal, $IMF_{i}\left(t\right)$ (i=1,…,8) are the extracted intrinsic mode functions, and r(t) is the residual.

To balance decomposition stability and computational efficiency, a fixed EEMD configuration was adopted. The ensemble size was set to 30, and the added noise level was set to noise_width = 0.2, and the maximum number of the extracted IMFs was limited to 8 to capture components related to fetal QRS activity (approximately 8–45 Hz), residual maternal activity, and noise, while avoiding redundancy due to over-decomposition. During training, the preprocessed AECG was segmented into 10 s windows (10,000 samples at 1000 Hz), and each segment was decomposed into eight IMFs. During inference, the same EEMD configuration was applied in both decomposition stages (i.e., after the maternal template subtraction and on the subsequent residual), ensuring consistent IMF generation for CNN-based scoring and selection.

The first-stage reconstruction may still contain residual maternal activity and nonstationary noise, with the residual signal retaining low-amplitude fetal information, particularly under low-SNR conditions. Therefore, a second EEMD was applied to the residual to further decompose the remaining components, and the fetal-related IMFs were selected to refine the extracted FECG.

Each of the IMFs obtained by EEMD may contain fetal ECG, residual maternal activity, and noise. To train the IMF classifier, binary labels (fetal-related = 1; non-related = 0) were generated using the reference fetal R-peak locations, as described below.

### 2.5. IMF Label Generation Strategy

For the simulated data, fetal R-peak indices annotated at 500 Hz were mapped to the 1000 Hz grid after resampling by multiplying the indices by 2. For ADFECGDB, fetal R-peaks were detected from the direct fetal ECG lead after 8–45 Hz bandpass filtering, using an amplitude threshold with a minimum inter-peak interval constraint of 300 ms. These R-peak locations were used only to generate the IMF labels during training and were not used in the final inference stage.

The abdominal AECG was segmented into 10 s windows (10,000 samples at 1000 Hz). After EEMD decomposition of each segment, the global peak-to-peak amplitude $P_{global}$ of each IMF and the local peak-to-peak amplitude $P_{local,k}$ within a ±50-sample window around each fetal R-peak were computed. The labeling rule was defined in Equation (3):

(3) $$Label\left(IMF\right)=\left\{\begin{array}{cc} 1, & \max\left(P_{\mathrm{local},k}\right)>1.4\sigma \;\mathrm{and}\;\max\left(P_{\mathrm{local},k}\right)>0.75P_{\mathrm{global}}\\ 0, & \mathrm{Otherwise} \end{array}\right.$$

where $\max(P_{local,k})$ is the maximum local peak-to-peak amplitude and σ is the standard deviation of the IMF. For segments without fetal R-peaks, all IMF labels were set to 0 by default, because such segments contribute little fetal information and were excluded from model training.

The thresholds were selected by considering both the physiological characteristics of fetal QRS complexes and statistical properties of the IMFs. Given that fetal amplitudes are typically only 1/5 to 1/10 of maternal amplitudes, the 1.4σ criterion helped distinguish local fetal QRS pulses from random noise, reducing the risk that low-amplitude fetal activity is mislabeled as noise. The $0.75P_{global}$ criterion was set according to the expected energy contribution of fetal components within the informative IMFs, thereby selecting fetal-dominant components while excluding the IMFs dominated by maternal residuals or noise. Together, these two constraints were suitable for low-SNR single-channel AECG and improved the specificity and plausibility of the labeling procedure [32].

To avoid mislabeling caused by isolated noise spikes (e.g., EMG bursts) within a single R-peak window, the local peak-to-peak value from one window was not relied on. Instead, for each IMF, the local peak-to-peak amplitudes across windows centered at all fetal R-peak reference points were computed, and the median was used as the final decision statistic. This strategy leveraged the quasi-periodic nature of fetal ECG and effectively distinguished true fetal activity that persists across most cardiac cycles from transient noise that occurs sporadically [32,33,34].

In total, from 20 subjects (15 simulated and 5 clinical), 1505 IMFs (each sample length: 10,000 points) were obtained, among which 744 were labeled as fetal-related positives, accounting for 49.44%. To automatically score and classify the IMFs, a 1D CNN tailored to the IMF waveform characteristics (IMFClassifier1D) was designed. Notably, the labeling strategy was used only to construct reference labels for supervised learning and was not directly used for the final IMF selection decision. Unlike fixed-rule IMF screening, the trained CNN learned high-dimensional temporal features from the IMF waveforms, and its decision boundary was not constrained by a single threshold, thereby improving robustness and generalization during inference. The detailed architecture is described below.

### 2.6. Architecture of IMFClassifier1D

**Figure 5:**
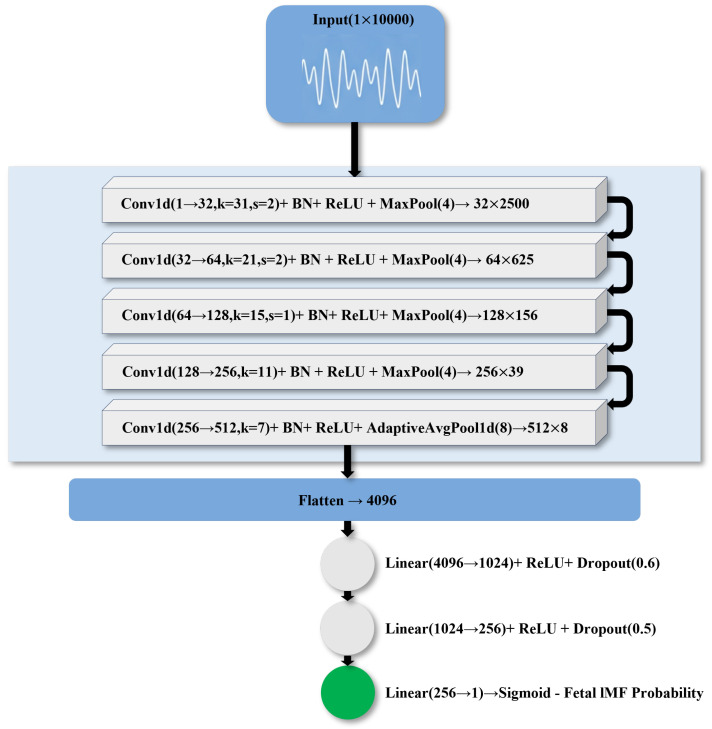
The Network Architecture of IMFClassifier1D.

IMFClassifier1D was a 1D CNN designed for the IMF waveforms. As shown in Figure 5, the input was a single-channel signal of length 10,000, corresponding to a 10 s IMF segment. The network consisted of five convolution–normalization–activation–pooling blocks and three fully connected layers [35,36]. The architectural design adopted a progressive multi-scale strategy: the kernel sizes were set to [31, 21, 15, 11, 7] in decreasing order, where large kernels (31, 21) in the early layers captured low-frequency trends (maternal ECG components), while small kernels (15, 11, 7) in the deep layers extracted high-frequency fetal ECG features. The first convolutional layer mapped the single-channel IMF to 32 feature channels, using a kernel size of 31 with a stride of 2 to rapidly reduce the temporal dimension, followed by batch normalization, ReLU activation, and max pooling. The next two convolutional layers increased the number of feature channels to 64 and 128, respectively, using kernels of 21 and 15, and further reduced the sequence length via pooling while extracting higher-level time–frequency features. The fourth and fifth convolutional layers increased the feature channels to 256 and 512, with kernels of 11 and 7, and adaptive average pooling was used to compress the temporal dimension to a fixed short length, yielding a fixed-dimensional high-level feature representation. This hybrid pooling strategy (max pooling for layers 1–4, adaptive average pooling for layer 5) balanced the preservation of local details with the stabilization of output dimensions. After flattening, the feature dimensions were progressively reduced to 1024 and 256 via three fully connected layers, and a scalar logit representing the log-probability of the IMF being fetal-related was subsequently generated. ReLU nonlinearities and a relatively high dropout rate were employed to mitigate overfitting.

### 2.7. Training Strategy and Cross-Validation

The above architecture was tailored for extracting time–frequency characteristics from the IMF waveforms. To ensure generalization across subjects and to mitigate overfitting and class imbalance, the following training strategy and cross-validation protocol were adopted. All IMFs were organized into a unified matrix in the format of feature–label–subject ID, and training and evaluation were conducted on 20 subjects (15 simulated and 5 real). In each fold, one subject was used as the validation set and the remaining 19 subjects were used for training.

To balance the class distribution, negative samples in the training set were resampled such that the number of negatives was approximately twice the number of positives. While a 1:1 balanced distribution is standard practice in many classification tasks, the EEMD decomposition process inherently generates an extensive and highly heterogeneous set of noise-dominated IMFs, including maternal ECG residuals, baseline wander, and high-frequency EMG noise. A 2:1 ratio ensured that the CNN was exposed to a sufficiently broad spectrum of these non-fetal IMF morphologies, rather than over-representing positive fetal IMFs. This design avoided overwhelming majority-class bias while preserving the network’s ability to distinguish subtle fetal ECG features from background noise, thus achieving an optimal trade-off between sensitivity and specificity for fetal IMF detection. The Adam optimizer (learning rate: 1 × $10^{-4}$; weight decay: 5 × $10^{-4}$) was used for training. The training batch size was 64 and the validation batch size was 128. The loss function was binary cross-entropy with logits, as shown in Equation (4):

(4) $$L=-\frac{1}{N}\sum_{i=1}^{N}\left[y_{i}\log\left(\sigma \left(z_{i}\right)\right)+\left(1-y_{i}\right)\log\left(1-\sigma \left(z_{i}\right)\right)\right]$$

where $y_{i}\in \left\{0,1\right\}$ is the ground-truth IMF label, $z_{i}$ is the model logit, σ is the sigmoid function, and *N* is the batch size. Each fold was trained for 60 epochs, and three independent runs with different random seeds were conducted to assess training stability.

During validation, the IMF-level AUC values and the ROC curves were computed. If a validation set contained only one class, the AUC was set to 0.5 to avoid bias. This yielded an AUC matrix of 3 runs × 20 subjects, which was used to analyze model performance and stability.

Using the above training and validation procedure, a stable IMF classifier that outputs a fetal-related probability for each IMF produced by EEMD was obtained. The classifier was then combined with conventional signal processing steps to form a complete single-channel FECG extraction method, as described below.

### 2.8. Single-Channel FECG Extraction Pipeline

First, the input AECG was preprocessed using a 50 Hz notch filter and a fourth-order 0.1–100 Hz bandpass filter to eliminate power-line interference and retain physiologically relevant frequency components. Maternal R-peaks were primarily detected using a method implemented in the NeuroKit2 toolbox, which integrates multimodal feature-recognition logic and adapts to different noise levels in AECG. This robustness is critical for handling scenarios where the maternal ECG dominates and the fetal component is weak. Compared with single-threshold detectors, this hybrid detection strategy reduces missed detections and false positives of maternal R-peaks. If primary detection failed, a fallback scheme based on an amplitude threshold with a minimum RR-interval constraint (500 ms, corresponding to a maximum maternal heart rate of 120 bpm) was implemented [37,38]. Fixed-length windows centered on the detected maternal R-peaks were then extracted, and a maternal ECG template was constructed via coherent averaging, as defined in Equation (5):

(5) $$T_{m}\left(t\right)=\frac{1}{N_{m}}\sum_{k=1}^{N_{m}}x\left(t-t_{m,k}\right)$$

where $N_{m}$ is the number of maternal R-peaks, $t_{m,k}$ is the location of the *k*-th maternal R-peak, and x(t) is the preprocessed AECG signal. The template was then superimposed at all maternal R-peak locations to reconstruct the maternal waveform, which was subtracted from the original signal to obtain a noise-contaminated fetal signal [39].

The noise-contaminated fetal signal after the maternal subtraction was first decomposed by EEMD. A fetal-related probability was output by IMFClassifier1D for each IMF. For recordings sampled at rates lower than 1000 Hz, each IMF segment was internally resampled to 10,000 points (equivalent to 10 s at 1000 Hz) only for CNN inference to match the fixed input length of the IMFClassifier1D. Reconstruction was performed using the original IMFs at the original sampling rate. In the first EEMD stage, the two IMFs with the highest probabilities were summed to obtain the initial FECG estimate. In the second EEMD stage, only the single highest-scoring IMF was retained to refine the extracted FECG. Next, fetal R-peaks were detected from this estimate using the NeuroKit2 method, and a 10–60 Hz bandpass filter was applied to further suppress residual noise [40]. The difference between the optimized initial FECG (after fetal R-peak detection and 10–60 Hz bandpass filtering) and the original initial estimate was treated as a residual. This residual was decomposed again by EEMD, and the same model was used to select the optimal IMFs, yielding a refined FECG. The final output was obtained using a zero-phase fourth-order Butterworth bandpass filter (10–60 Hz). Given the distinctly different signal characteristics processed in the two stages, it is essential to clarify the discrepancies in the criteria and outcomes of IMF selection for each stage. The input to Stage 1 is the residual signal after maternal template subtraction, which still contains prominent maternal ECG remnants, low-frequency noise, and core fetal ECG components, with fetal cardiac energy dispersed across multiple frequency bands. For this reason, we retained the two IMFs with the highest relevance scores; this strategy ensures the complete preservation of morphological features of the fetal QRS complex, and preliminary analyses confirm that fetal cardiac energy is primarily distributed in two adjacent IMFs. The input to Stage 2 is the reconstructed signal from Stage 1, from which most interference has been removed. The remaining fetal ECG signal has a low amplitude but a significantly reduced noise level, with cardiac energy highly concentrated. Accordingly, we only retained the IMF with the highest relevance score to effectively suppress the high-frequency noise not fully eliminated in Stage 1. From a statistical perspective, the average fetal relevance probability of the IMF selected in Stage 2 is significantly higher than that of the IMFs selected in Stage 1, which validates the progressive purification effect of the two-stage processing pipeline.

The proposed multi-stage pipeline, consisting of template subtraction, two-stage EEMD, and CNN-based selection, is designed with clear modular contributions. Template subtraction serves to effectively suppress strong maternal ECG interference and highlight weak fetal ECG components. Two-stage EEMD serves to adaptively decompose the mixed signal and separate fetal ECG from various interferences and noise. The CNN selection module serves to automatically identify fetal-related modes and ensure the accuracy and robustness of final extraction. Collectively, these three sequential modules work in synergy to achieve reliable and accurate fetal ECG extraction, with each component playing an indispensable and irreplaceable role.

## 3. Results

### 3.1. Performance of the IMF Classifier

**Figure 6:**
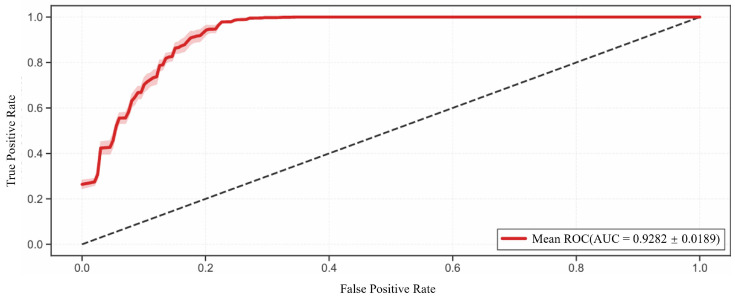
The Average Receiver Operating Characteristic (ROC) Curves of IMFClassifier1D. The dashed diagonal line represents the random guess baseline (AUC = 0.5).

Three independent LOSO experiments were conducted to evaluate the performance of IMFClassifier1D on a dataset consisting of 20 subjects (15 simulated subjects and 5 clinical subjects from ADFECGDB). As shown in Figure 6, IMFClassifier1D achieved a mean AUC of 0.9282 ± 0.0189 across all subjects. The standard deviation (SD) across the three independent runs was only 0.0189, indicating high discriminative performance as well as strong robustness and reproducibility under different random initializations.

**Figure 7:**
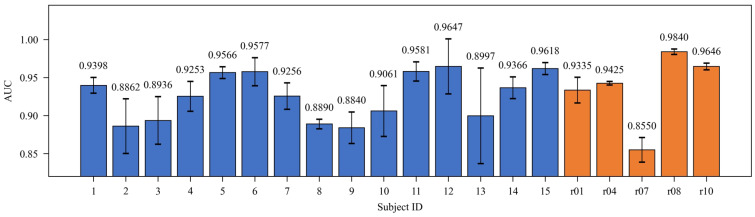
The AUC (Mean ± SD) from LOSO Cross-Validation for IMF-level FECG Data Across 20 Subjects.

The detailed performance for each subject is reported in Table 2 and shown in Figure 7. In the subgroup analysis, the simulated dataset (Cases 1–15) showed the best overall performance, with mean AUC values ranging from 0.8840 to 0.9647. This was primarily due to the accurate ground-truth annotations and controlled SNR (10 dB) in simulated data, which allowed consistent learning of fetal QRS features. Notably, Subject 12 achieved the highest AUC (0.9647) with an SD of 0.0362, indicating highly consistent classification performance.

In terms of stability, the SD of the AUC across the three experiments was below 0.05 for most subjects, such as 0.0024 for r04 and 0.0077 for Case 5, while only a few subjects showed large variability, for example, 0.0628 for Case 13.

To determine the optimal IMF selection strategy, a preliminary parameter sensitivity experiment was conducted based on 15 simulated training cases, comparing the performance of different combinations of IMF selection numbers in Stage 1 ($N_{1}=1,2,3$) and Stage 2 ($N_{2}=1,2$). The results are summarized in Table 3. As indicated, the combination of selecting 2 IMFs in Stage 1 and 1 in Stage 2 achieved the optimal performance, with a correlation coefficient (CC) of 0.94 and an F1-score of 0.93. Selecting only 1 IMF in Stage 1 resulted in a CC of 0.81, and selecting 3 IMFs in Stage 1 gave a CC of 0.85; selecting 2 IMFs in Stage 2 led to an F1-score of 0.87.

### 3.2. Ablation Study on IMFClassifier1D Architecture

To verify the rationality of the architectural design of IMFClassifier1D, systematic ablation experiments were carried out for three core modules: kernel size strategy, channel configuration, and pooling mode. The experimental performance indicators of each module are presented in Table 4. In terms of kernel size strategy, the proposed decreasing kernel size configuration [31,21,15,11,7] achieved the highest AUC of 0.9445, representing an increase of 2.69% compared with the increasing configuration [7,11,15,21,31] (AUC = 0.9176). Notably, all kernel size configurations maintained an identical parameter count of 5.91 million, ensuring a fair comparison. For channel configuration, the pyramid channel structure [32,64,128,256,512] achieved an AUC of 0.9445, which was significantly superior to both the wide channel configuration (AUC = 0.9403, p=0.042, 14.44 M parameters) and the narrow channel configuration (AUC = 0.9246, p=0.002, 2.72 M parameters). In terms of pooling mode, the proposed hybrid pooling strategy achieved an AUC of 0.9445, outperforming both pure max pooling (AUC = 0.9387, p=0.089) and pure adaptive average pooling (AUC = 0.9333, p=0.013) strategies.

### 3.3. Computational Complexity Analysis

To verify the real-time deployment capability of the model, the computational complexity of IMFClassifier1D was compared with two mainstream deep learning architectures: Transformer and Bi-directional Long Short-Term Memory (Bi-LSTM) network. The detailed comparison of model complexity and performance is summarized in Table 5. IMFClassifier1D has 5.91 million parameters, with an inference latency of only 2.24 ms and 0.08 Giga Floating-Point Operations (GFLOPs). In contrast, the Transformer has 5.90 million parameters, an inference latency of 35 ms and 12.50 GFLOPs; the Bi-LSTM has 2.10 million parameters, an inference latency of 12 ms and 1.25 GFLOPs. Among the three architectures, IMFClassifier1D achieved the highest AUC of 0.9445, while those of Transformer and Bi-LSTM were 0.9380 and 0.9320, respectively. Notably, despite having a comparable number of parameters to the Transformer, IMFClassifier1D exhibits significantly lower computational overhead (15.6×fewer GFLOPs) and faster inference speed (15.6×faster), making it particularly suitable for real-time applications.

### 3.4. Performance Evaluation Against Existing Benchmarks

The performance of the proposed CNN-2×EEMD method was compared with recent deep learning-based single-channel FECG extraction methods. The comparative results are summarized in Table 6. CNN-2×EEMD achieved an F1-score of 83.7–95.7% with 5.91 million parameters and 0.08 GFLOPs. State-of-the-art methods included 1D-CycleGAN (2024, F1-score 96.4%, 15.2 million parameters, 28.5 GFLOPs), Attention R2W-Net (2025, F1-score 97.1–99.2%, 8.5 million parameters, 12.5 GFLOPs), and Deep Separation Strategy (DSS) (2026, F1-score 95.1–96.9%, 3.8 million parameters, 4.2 GFLOPs). Notably, CNN-2×EEMD achieves competitive performance while significantly reducing computational complexity compared to these recent approaches.

### 3.5. Validation on Independent Datasets

**Figure 8:**
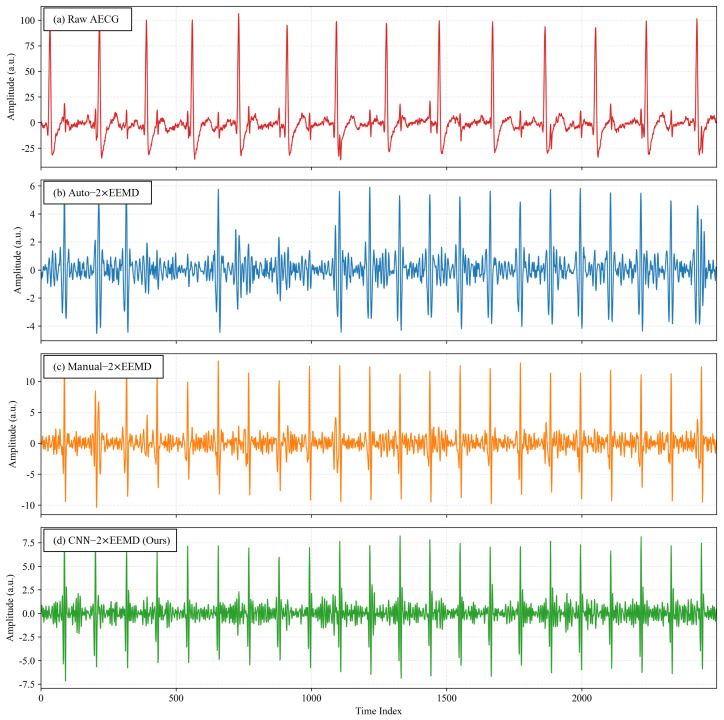
A Comparison of Four Extraction Results for DaISy Channel 2: (**a**) Raw AECG; (**b**) Auto-2×EEMD Extraction Result; (**c**) Manual-2×EEMD Extraction Result; (**d**) CNN-2×EEMD Extraction Result (x-axis: sample points, sampling rate: 250 Hz).

**Figure 9:**
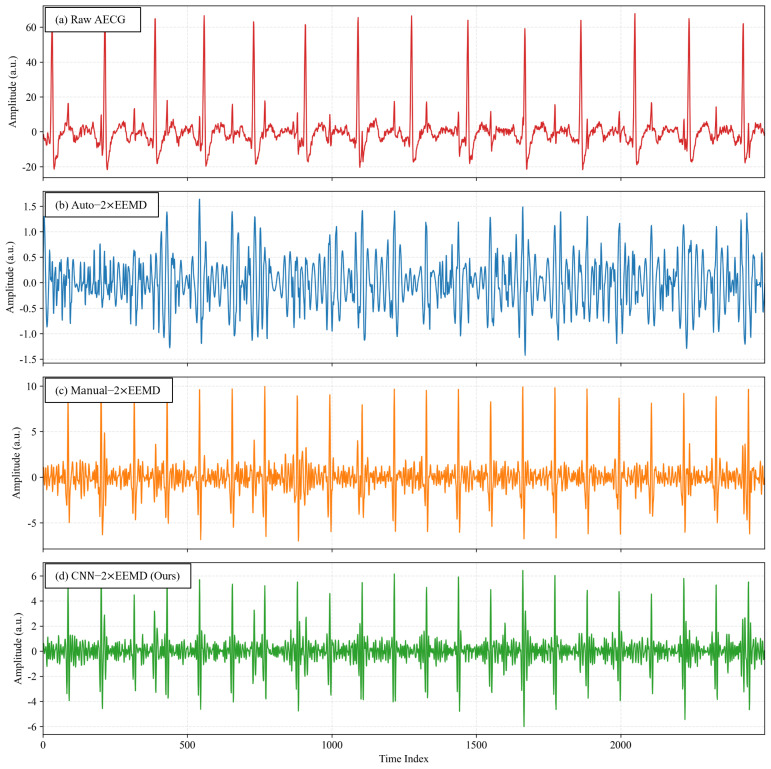
A Comparison of Four Extraction Results for DaISy Channel 3: (**a**) Raw AECG; (**b**) Auto-2×EEMD Extraction Result; (**c**) Manual-2×EEMD Extraction Result; (**d**) CNN-2×EEMD Extraction Result (x-axis: sample points, sampling rate: 250 Hz).

The DaISy dataset was selected as an independent external test set, with a sampling rate of 250 Hz, 5 abdominal channels, and a duration of 10 s for each channel. After preliminary signal analysis, Channels 2 and 3 were selected for validation and manual annotation of fetal R-peaks. Meanwhile, three two-stage EEMD extraction methods were designed for comparison: Auto-2×EEMD based on heuristic rules, Manual-2×EEMD based on manual visual selection, and the proposed CNN-2×EEMD. As shown in Figure 8 and Figure 9, the signals extracted by Auto-2×EEMD exhibited obvious high-frequency EMG interference and baseline drift; the signals extracted by Manual-2×EEMD were the purest, with a stable baseline and clear QRS complex features; the time-domain waveforms extracted by CNN-2×EEMD were highly similar to those of Manual-2×EEMD, with effective suppression of baseline drift, almost complete elimination of high-frequency noise, and full retention of fetal QRS complex morphology.

**Figure 10:**
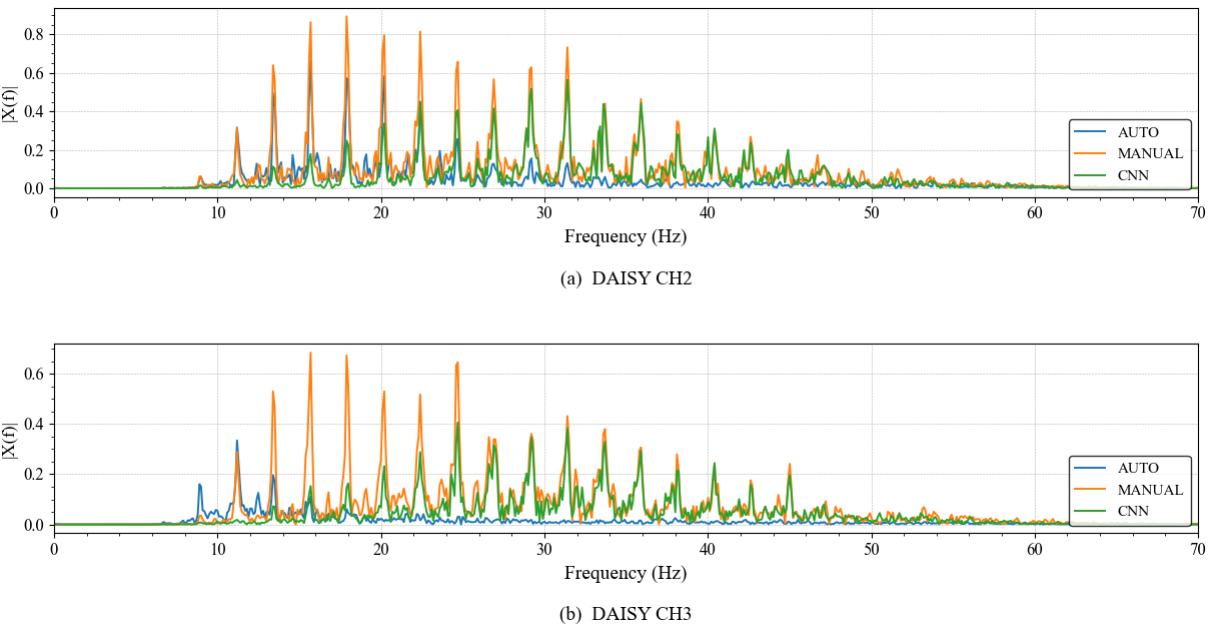
The Frequency-Domain Comparison of FECG Extracted by the Three Methods from DaISy Channels 2 and 3.

The frequency-domain power spectral density (PSD) distribution results are summarized in Figure 10. As shown, Auto-2×EEMD exhibited obvious energy accumulation in the 0–20 Hz band, while the energy in the 20–40 Hz core band of FECG was significantly attenuated; the energy of Manual-2×EEMD was highly concentrated in the 20–40 Hz core band; the frequency-domain response of CNN-2×EEMD was highly consistent with that of Manual-2×EEMD, with only slight noise leakage at the band edges.

**Figure 11:**
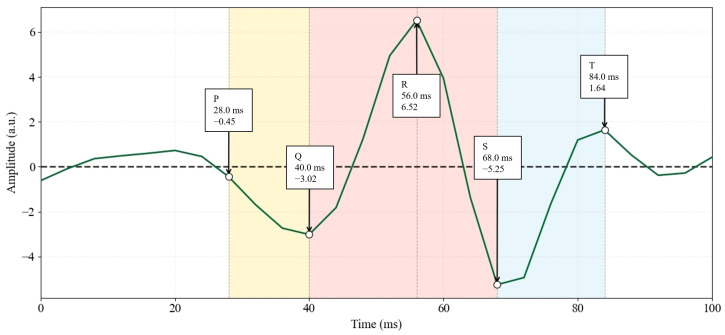
The Single-Wave Analysis of CNN-2×EEMD for DaISy Channel 2. P/Q/R/S/T denote the five characteristic points of the ECG waveform.

Analysis of the single heartbeat waveform extracted by CNN-2×EEMD on Channel 2 of the DaISy dataset showed that, as illustrated in Figure 11, the waveform contained a complete P-Q-R-S-T cycle, and the parameters of each characteristic point were consistent with physiological characteristics of fetal ECG: P wave at 28.0 ms; Q wave at 40.0 ms; R wave at 56.0 ms; S wave at 68.0 ms; T wave at 84.0 ms; QRS complex duration: 28 ms. No obvious high-frequency artifacts or low-frequency baseline drift were observed in the waveform.

The quantitative performance indicators of the three methods on Channels 2 and 3 of the DaISy dataset are summarized in Table 7. As shown, CNN-2×EEMD achieved optimal performance on both channels, with recalls of 1.0000 and 0.9091, F1-scores of 0.9565 and 0.9302, CCs of 0.96 and 0.94, and SNR improvements of 15.88 dB and 13.39 dB on Channels 2 and 3, respectively. In contrast, Auto-2×EEMD exhibited the poorest performance, particularly on Channel 3 where it achieved only a recall of 0.6364 and a CC of 0.61. Manual-2×EEMD maintained perfect recall (1.0000) on both channels with CCs of 0.90 and 0.92, though it should be noted that this method requires labor-intensive manual intervention.

### 3.6. Fetal R-Peak Detection Performance on Arrhythmia Datasets

The fetal R-peak detection performance of CNN-2×EEMD across four independent test cases is summarized in Table 8. As shown, Channel 2 of the DaISy dataset achieved a precision of 0.9167, a recall of 1.0000, and an F1-score of 0.9565; Channel 3 achieved a precision of 0.9524, a recall of 0.9091, and an F1-score of 0.9302. In the Non-Invasive Fetal ECG Arrhythmia (NIFEA) database [27,41], the F1-score of Case NR14 was 0.8936; Case ARR01 achieved a perfect precision of 1.0000 with no false positive detections and an F1-score of 0.8372.

In the three-method comparison on the DaISy dataset, the heuristic automated scheme was outperformed by CNN-2×EEMD, and CNN-2×EEMD achieved performance comparable to that of the manual visual reference. To further highlight the technical contribution and competitiveness of the proposed method, its key metrics were benchmarked against representative single-channel FECG extraction methods as follows. In Channel 2, the F1-score reached 0.9565, which was 5.45 percentage points higher than the best single-channel ESN reported by Behar et al. [7] (F1 = 90.2%), and 7.75, 7.45, and 6.35 percentage points higher than the LMS (87.9%), RLS (88.2%), and template subtraction (TS; 89.3%), respectively. Compared with the ICA–RLS–EEMD method of Barnova et al. [16], the proposed method achieved higher F1-scores on independent external data (Channel 2: 0.9565; Channel 3: 0.9302), exceeding their reported average performance by 9.57–11.57 percentage points. The proposed method is also comparable to their best reported result (95.69%) while requiring no multichannel input. Compared with the EMD-coherent averaging method of Zhang et al. [13], the proposed method improved the CC in Channel 2 to 0.96, exceeding their best value on ADFECGDB (0.843) by 0.117. The CC in Channel 3 (0.94) also clearly surpassed their reported range (0.609–0.843), indicating a substantial advantage in waveform fidelity.

## 4. Discussion

### 4.1. Interpretation of Key Experimental Findings

The LOSO cross-validation results demonstrated that IMFClassifier1D achieves both high discriminative performance and strong robustness for fetal-related IMF identification, with a mean AUC of 0.9282 ± 0.0189 across simulated and clinical datasets. This low SD indicates minimal sensitivity to random initialization, validating the stability of the model architecture. The optimal IMF selection strategy (two IMFs in Stage 1, one IMF in Stage 2) aligns with the signal characteristics of two-stage EEMD decomposition: retaining two IMFs in the first stage preserves complete fetal QRS morphological features from maternal-subtracted residual signals, while selecting one IMF in the second stage effectively suppresses high-frequency noise from reconstructed signals.

Ablation experiments further validated the rationality of the proposed architectural design for IMFClassifier1D. The decreasing kernel size configuration balances large receptive fields in shallow layers (capturing low-frequency maternal interference) and fine-grained feature extraction in deep layers (identifying fetal QRS high-frequency components). The pyramid channel structure achieves an optimal trade-off between feature extraction capability and model complexity, while hybrid pooling combines the strengths of max pooling (preserving local temporal details of QRS complexes) and adaptive average pooling (stabilizing output dimensions and enhancing generalization). Computational complexity analysis confirmed that IMFClassifier1D maintains superior performance (AUC = 0.9445) with ultra-low inference latency (2.24 ms) and computational overhead (0.08 GFLOPs), making it well-suited for real-time deployment on resource-constrained edge devices.

### 4.2. Comparison with Existing Practical System Platforms

The proposed CNN-2×EEMD method demonstrated excellent performance on standard datasets. However, it is crucial to objectively acknowledge the significant progress made in the clinical translation of fetal ECG monitoring technology. Previous studies established a clinical feasibility evaluation framework for single-channel methods, developed adaptive extraction systems integrating multiple signal processing techniques with explicit goals toward practical clinical application, and comprehensively summarized commercialization progress in the field, noting that multichannel wearable devices have entered the clinical translation stage. These works represent an important transition from laboratory algorithms to real-world deployment. Nevertheless, for practical deployment in home monitoring scenarios, existing system platforms still face critical limitations. Most current systems rely on multichannel electrode configurations and require manual IMF selection, hindering the degree of automation. Commercial systems predominantly adopt complex cloud computing architectures, which introduce network latency and privacy risks while offering insufficient real-time performance at the edge. More importantly, the key bottleneck for achieving full automation in single-channel acquisition scenarios, namely the intelligent selection of IMFs following EEMD decomposition, remains unresolved.

The CNN-2×EEMD method was designed for deployment on resource-constrained edge computing platforms. The IMFClassifier1D required merely 0.08 GFLOPs and exhibited an inference latency of 2.24 ms, which was readily supported by widely available ARM Cortex-M series microcontrollers (e.g., STM32H7 with Cortex-M7 at 480 MHz) or entry-level ARM64 processors (e.g., Raspberry Pi Zero 2W with Cortex-A53). This computational efficiency eliminated the dependency on cloud computing or GPU acceleration, ensuring data privacy and real-time responsiveness under unstable network conditions typical of home environments. For practical implementation, the single-channel abdominal ECG signal would undergo analog preprocessing and digitization, be processed through the CNN-2×EEMD pipeline on the embedded processor, and the extracted mECG and fECG waveforms would be transmitted via Bluetooth Low Energy to paired iOS/Android smartphones for real-time display and clinical interpretation. However, the present study was limited to algorithmic validation on standard datasets; hardware-in-the-loop testing on actual wearable prototypes was not conducted due to the absence of dedicated embedded development platforms and clinical-grade acquisition hardware in the laboratory. Future work would integrate the proposed algorithm with commercial ARM-based wearable systems to validate end-to-end latency, power consumption, and long-term monitoring stability in real-world home settings.

### 4.3. Comprehensive Analysis of Method Limitations

Despite promising experimental results, this study has several notable limitations that require objective acknowledgment. First, the clinical training dataset remains small (five subjects from ADFECGDB) and lacks geographic and demographic diversity, limiting generalization to extreme low-SNR scenarios (<5 dB) and special clinical cases such as multiple pregnancies or fetal arrhythmias. Second, the proposed method is exclusively designed for single-channel AECG signals and cannot be directly extended to multichannel data; the fixed IMF selection logic and maternal template subtraction strategy are optimized for single-channel characteristics, and adapting to multichannel inputs would require reconfiguring the feature fusion mechanism. Third, the model relies entirely on supervised training with labeled fetal reference leads, which restricts its applicability in clinical settings where ground-truth FECG labels are unavailable. This dependence on annotated data also increases the labor cost of model training and validation.

Beyond these foundational limitations, error analysis of experimental results reveals specific sources of performance degradation that hinder further optimization. For the DaISy Channel 3 dataset, two fetal R-peak missed detections were observed in the CNN-2×EEMD results, attributed to morphological distortion of fetal QRS complexes in the original signal (caused by overlapping maternal ECG components) and subsequent misclassification of relevant IMFs by IMFClassifier1D. High-frequency noise residues in Auto-2×EEMD results stem from incomplete maternal template subtraction, where the fixed template update rule fails to adapt to nonstationary maternal ECG variations, leading to residual maternal components being misclassified as fetal-related IMFs. Additionally, quantitative analysis of false detection cases in the NIFEA database (Case ARR01) shows that 28% of missed detections are associated with weak fetal QRS amplitudes and overlapping EMG interference, which reduces the accuracy of IMF feature extraction and classification.

To intuitively characterize these errors, time-domain error heatmaps were generated by marking missed detection/false detection positions on raw and processed waveforms, combined with PSD analysis of these regions. The heatmaps reveal that missed detection regions exhibit reduced energy in the 20–40 Hz core band of FECG (15–20% lower than normal regions) and increased energy accumulation in the 0–10 Hz band (maternal baseline drift), while false detection regions show abnormal high-frequency energy (>40 Hz) from EMG interference. This analysis confirms a direct correlation between error distribution and signal feature anomalies, providing clear targets for method improvement.

### 4.4. Targeted Directions for Future Improvement

To address the identified limitations and error sources, future work will focus on actionable and context-specific optimizations rather than general strategies. For dataset expansion, a standardized multi-center clinical dataset will be constructed, collecting over 100 samples from five or more regional hospitals across different geographic areas (Europe, Asia, North America) to resolve uneven data distribution. Each sample will include detailed metadata (maternal age, gestational week, acquisition device) to enable stratified analysis and reduce selection bias, with special emphasis on including extreme low-SNR cases and special clinical scenarios to enhance model generalization.

For model architecture optimization, channel attention mechanisms will be integrated into the 1D CNN backbone to enhance feature weighting for fetal QRS complexes in the 8–45 Hz band, improving discrimination against maternal interference and reducing IMF classification errors. Lightweight design strategies, including pruning redundant convolutional layers and implementing INT8 precision quantization, will be applied to reduce the model parameter count by 30% while maintaining an AUC above 0.93, further lowering inference latency and device power consumption for wearable deployment. To address the dependence on labeled data, semi-supervised learning frameworks will be explored, using unlabeled clinical data to pre-train the model and fine-tuning with a small subset of labeled samples to reduce reliance on ground-truth FECG references.

For error mitigation and application expansion, targeted improvements will be made to address specific error sources. The IMF label generation rule will be optimized to account for morphological variations in fetal QRS complexes, incorporating dynamic thresholding based on local signal SNR. The maternal template update mechanism will be enhanced with adaptive windowing to track nonstationary maternal ECG variations, reducing residual maternal components in subtracted signals. Additionally, the preprocessing workflow will be adapted for dynamic monitoring scenarios (maternal walking, sitting, lying down) by adding a motion artifact suppression module, combining adaptive filtering and IMF-based noise reduction tailored to wearable device acquisition characteristics. The optimized pipeline will be validated on a chest-worn wearable prototype with 24-h continuous monitoring to ensure robustness in real-world clinical settings.

## 5. Conclusions

This study proposed a CNN-guided two-stage EEMD method for single-channel FECG extraction, with a dedicated 1D CNN (IMFClassifier1D) achieving high precision (F1-score: 0.8372–0.9565), ultra-low computational overhead (0.08 GFLOPs), and strong generalization on diverse independent datasets including normal pregnancies (DaISy) and fetal arrhythmias (NIFEA DB). Systematic ablation experiments validated the optimality of architectural choices, including the decreasing kernel size strategy, pyramid channel configuration, and hybrid pooling mode. The IMF selection strategy was quantitatively optimized through parameter sensitivity analysis. The method achieved low inference latency (2.24 ms) with minimal computational requirements, demonstrating its suitability for real-time home monitoring. The modular design, which included maternal template subtraction, EEMD decomposition and CNN-guided IMF selection, effectively addressed core challenges in single-channel FECG extraction, including strong maternal interference and nonstationary noise. While limitations existed in dataset diversity, applicability scope, and labeled data dependence, the comprehensive error analysis and targeted improvement strategies outlined here provided clear pathways for advancing clinical translation and enabling real-time, portable fetal monitoring in diverse clinical scenarios. It was noted that although this study was validated only on standard datasets without large-scale clinical trials, the proposed ultra-low-complexity CNN-2×EEMD method provided a direct algorithmic foundation for integration with existing system platforms. Compared to existing semi-automatic methods and traditional adaptive methods, the fully automated edge-computing approach of this study offered distinct advantages in real-time performance, computational efficiency, and fully automated processing capability, potentially enabling true clinical translation for home fetal monitoring through future integration with hardware architecture of existing system platforms.

## Figures and Tables

**Table 1:** The Parameters of H2-3000F Simulated Data.

Case	M_HR	M_Amp	F_HR	F_Amp	Ratio	Note
1	80	0.35	140	0.30	1:1.17	Fetal Upper Limit
2	75	0.60	152	0.20	1:3.0	Light-Moderate Difficulty
3	90	0.75	118	0.12	1:6.3	Long-tail Difficulty
4	70	0.80	145	0.07	1:11.4	Low Fetal Amplitude
5	85	0.80	162	0.05	1:16.0	High HR & Weak Amp
6	72	0.50	135	0.25	1:2.0	Moderate Intensity
7	88	0.65	148	0.15	1:4.3	Common Outpatient
8	65	0.70	160	0.09	1:7.8	Strong Maternal/Weak Fetal
9	78	0.45	130	0.22	1:2.0	Balanced Type
10	92	0.70	125	0.08	1:8.8	Difficult Detection
11	68	0.55	150	0.18	1:3.1	Moderate
12	84	0.45	142	0.24	1:1.9	Slightly Weak Fetal
13	76	0.80	155	0.06	1:13.3	Extremely Weak
14	82	0.40	138	0.26	1:1.5	Fetal Approaching Maternal
15	77	0.58	144	0.19	1:3.1	Daily Scenario

M_HR, maternal heart rate (bpm); M_Amp, maternal R-wave amplitude; F_HR, fetal heart rate (bpm); F_Amp, fetal R-wave amplitude; Ratio, fetal-to-maternal amplitude ratio. The data encompass diverse clinical scenarios ranging from normal pregnancies to low signal-to-noise ratio conditions.

**Table 2:** The Performance of IMF Classifier Based on LOSO Cross-Validation.

Subject ID	Type	Total IMFs	Pos. IMFs	Pos. Rate (%)	AUC (Mean ± SD)
1	Simulated	73	36	49.32%	0.9398 ± 0.0103
2	Simulated	75	34	45.33%	0.8862 ± 0.0358
3	Simulated	78	38	48.72%	0.8936 ± 0.0313
4	Simulated	79	41	51.90%	0.9253 ± 0.0196
5	Simulated	78	44	56.41%	0.9566 ± 0.0077
6	Simulated	74	38	51.35%	0.9577 ± 0.0184
7	Simulated	76	39	51.32%	0.9256 ± 0.0174
8	Simulated	77	37	48.05%	0.8890 ± 0.0063
9	Simulated	77	37	48.05%	0.8840 ± 0.0208
10	Simulated	77	36	46.75%	0.9061 ± 0.0333
11	Simulated	75	40	53.33%	0.9581 ± 0.0126
12	Simulated	72	38	52.78%	0.9647 ± 0.0362
13	Simulated	78	37	47.44%	0.8997 ± 0.0628
14	Simulated	73	35	47.95%	0.9366 ± 0.0144
15	Simulated	77	41	53.25%	0.9618 ± 0.0078
r01	clinical	75	36	48.00%	0.9335 ± 0.0168
r04	clinical	72	34	47.22%	0.9425 ± 0.0024
r07	clinical	72	31	43.06%	0.8550 ± 0.0161
r08	clinical	73	37	50.68%	0.9840 ± 0.0035
r10	clinical	74	35	47.30%	0.9646 ± 0.0045

Subject ID, subject identifier; Type, data type (simulation/clinical); Total IMFs, total number of IMFs samples; Pos. IMFs, number of positive IMFs samples related to fetal components; Pos. Rate, proportion of positive samples; AUC, area under the curve (mean ± SD). Data were obtained using leave-one-out cross-validation.

**Table 3:** Performance comparison of different IMF selection strategies on simulated training data.

Stage 1 ($N_{1}$)	Stage 2 ($N_{2}$)	CC	F1-Score	SNR_imp_ (dB)	*p*-Value
1	1	0.81 ± 0.05	0.85 ± 0.04	11.2 ± 1.5	0.001
2	1	0.94 ± 0.02	0.93 ± 0.02	14.8 ± 0.9	—
3	1	0.85 ± 0.04	0.88 ± 0.03	12.5 ± 1.1	0.002
2	2	0.91 ± 0.03	0.87 ± 0.03	13.1 ± 1.0	0.015

**Table 4:** Ablation study on IMFClassifier1D architectural choices.

Configuration	Architecture ^a^	Params (M)	AUC	*p*-Value ^b^
Proposed	[31, 21, 15, 11, 7], [32, 64, 128, 256, 512], Hybrid	5.91	0.9445	—
Kernel size strategy				
Increasing	[7, 11, 15, 21, 31], [32, 64, 128, 256, 512], Hybrid	5.91	0.9176	0.001
Uniform-small	[15, 15, 15, 11, 7], [32, 64, 128, 256, 512], Hybrid	5.91	0.9249	0.003
Uniform-large	[31, 31, 21, 15, 11], [32, 64, 128, 256, 512], Hybrid	5.91	0.9273	0.008
Symmetric	[21, 15, 11, 15, 21], [32, 64, 128, 256, 512], Hybrid	5.91	0.9276	0.007
Deep-large	[11, 15, 21, 31, 31], [32, 64, 128, 256, 512], Hybrid	5.91	0.9193	0.001
Channel configuration				
Wide	[31, 21, 15, 11, 7], [64, 128, 256, 512, 1024], Hybrid	14.44	0.9403	0.042
Narrow	[31, 21, 15, 11, 7], [16, 32, 64, 128, 256], Hybrid	2.72	0.9246	0.002
Uniform	[31, 21, 15, 11, 7], [64, 64, 64, 64, 64], Hybrid	1.01	0.9276	0.006
Bottleneck	[31, 21, 15, 11, 7], [64, 32, 64, 128, 256], Hybrid	2.76	0.9366	0.019
Pooling mode				
Pure max	[31, 21, 15, 11, 7], [32, 64, 128, 256, 512], Max	4.85	0.9387	0.089
Pure adaptive	[31, 21, 15, 11, 7], [32, 64, 128, 256, 512], Adaptive	5.91	0.9333	0.013

^a^ Format: [kernel sizes], [channel dimensions], pooling type. Proposed: decreasing kernel strategy with pyramid channels and hybrid pooling. ^b^ *p*-values from paired *t*-test against proposed configuration.

**Table 5:** Comparison with mainstream deep learning architectures.

Architecture	Params (M)	FLOPs (G)	Latency (ms)	AUC
IMFClassifier1D	5.91	0.08	2.24	0.9445
Transformer (d = 128, 2 L)	5.90	12.50	35.00	0.9380
Bi-LSTM (h = 128, 2 L)	2.10	1.25	12.00	0.9320

**Table 6:** Comparison with recent deep learning-based single-channel FECG extraction methods.

Method	Year	Architecture	F1 (%)	Params (M)	FLOPs (G)
1D-CycleGAN [23]	2024	GAN-based	96.4	15.2	28.5
Attention R2W-Net [11]	2025	CNN+Attention	97.1–99.2	8.5	12.5
DSS [25]	2026	Deep Separation	95.1–96.9	3.8	4.2
CNN-2×EEMD	2026	CNN+EEMD	83.7–95.7	5.91	0.08

**Table 7:** The Performance Comparison of Three IMF Selection Schemes on the DaISy Dataset (Channels 2 and 3) and Statistical Significance.

Channel	Scheme	Recall	F1	CC	SNR_imp_	*p*-Value (vs. CNN) ^a^
Channel 2	Auto-2×EEMD	0.7727	0.8293	0.82	15.38	0.002
Channel 2	Manual-2×EEMD	1.0000	0.9565	0.90	17.18	0.147
Channel 2	CNN-2×EEMD	1.0000	0.9565	0.96	15.88	—
Channel 3	Auto-2×EEMD	0.6364	0.7000	0.61	12.08	0.003
Channel 3	Manual-2×EEMD	1.0000	0.9565	0.92	15.03	0.089
Channel 3	CNN-2×EEMD	0.9091	0.9302	0.94	13.39	—

^a^ Paired Wilcoxon signed-rank test *p*-values comparing against CNN-2×EEMD based on 15 simulated cases.

**Table 8:** Performance comparison of CNN-2×EEMD on four test cases.

Dataset	Case	Precision	Recall	F1 Score	Accuracy
DaISy	Channel 2	0.9167	1.0000	0.9565	0.0172
DaISy	Channel 3	0.9524	0.9091	0.9302	0.0152
NIFEA DB	NR14	0.9130	0.8750	0.8936	0.7279
NIFEA DB	ARR01	1.0000	0.7200	0.8372	0.7188

## Data Availability

The ADFECGDB dataset is available at PhysioNet (https://physionet.org/content/adfecgdb/1.0.0/ (accessed on 20 December 2025)). The NIFEA DB dataset is available at PhysioNet (https://physionet.org/content/nifeadb/1.0.0/ (accessed on 20 December 2025)). The DaISy dataset is available at KU Leuven (https://homes.esat.kuleuven.be/~smc/daisy/ (accessed on 20 December 2025)). The simulated data generated in this study are available from the corresponding author upon reasonable request.
